# Luteolin attenuates LPS-induced damage in IPEC-J2 cells by enhancing mitophagy via AMPK signaling pathway activation

**DOI:** 10.3389/fnut.2025.1552890

**Published:** 2025-03-26

**Authors:** Jianyun Yuan, Ke Zhang, Lingling Yang, Xinyi Cheng, Jinyan Chen, Xiaoquan Guo, Huabin Cao, Caiying Zhang, Chenghong Xing, Guoliang Hu, Yu Zhuang

**Affiliations:** Jiangxi Provincial Key Laboratory for Animal Health, Institute of Animal Population Health, College of Animal Science and Technology, Jiangxi Agricultural University, Nanchang, Jiangxi, China

**Keywords:** luteolin, lipopolysaccharide, AMPK, IPEC-J2 cells, mitophagy

## Abstract

**Background:**

Luteolin (LUT), a flavonoid compound widely present in natural plants, has been extensively studied for its diverse biological properties, involving anti-inflammatory,antioxidant, anti-apoptosis and other properties.

**Methods:**

The aim of this study was to investigate the effect of LUT on lipopolysaccharide (LPS)-induced Intestinal Porcine Epithelial Cell line–J2 (IPEC-J2 cells) damage and its underlying mechanism.

**Results:**

The experiment showed that LPS treatment induced injury in IPEC-J2 cells, leading to tight junction disruption, ROS accumulation, and cell apoptosis. Remarkably, LUT attenuated LPS-induced IPEC-J2 cells damage by the up-regulation of Zonula Occludens–1(ZO-1), Occludin, and Claudin protein 1 (Claudin-1) protein expression levels.Besides, LUT increased the activities of CAT, and SOD and prevented LPS-induced MDA and ROS production. LUT suppressed Nuclear Factor kappa-light-chain-enhancer of activated B cells (NF-κB) activation in LPS-induced IPEC-J2 cells, reducing (Interleukin-1beta) IL-1β and Interleukin–6 (IL-6) expression. Moreover, LUT attenuated LPS-induced apoptosis in IPEC-J2 cells by up-regulating expression of B-cell lymphoma 2 (Bcl-2) and down-regulating expression of Cysteine-aspartic acid protease 3 (Caspase-3), Cysteine - aspartic acid protease 9 (Caspase-9) and Bcl-2-associated X protein (Bax). Furthermore, LUT upregulated the AMP–activated protein kinase (AMPK)/Unc–51 like autophagy activating kinase (ULK) signaling pathway and Parkin–RBR E3 ubiquitin protein ligase (Parkin)/PTEN induced putative kinase 1 (PINK1)–mediated mitophagy in a dose–dependent manner. When AMPK was knocked down by short–hairpin RNA (shRNA), the protective effects of LUT against LPS–induced IPEC–J2 cell damage were weakened, as evidenced by the accumulation of excessive ROS and impaired mitophagy.

**Conclusion:**

In summary, LUT exhibits the ability to protect against LPS-induced damage to intestinal tight junctions by enhancing mitophagy through AMPK activation.

## 1 Introduction

Intestinal epithelial cells (IECs) play a pivotal role in fundamental physiological processes, including food digestion, nutrient absorption, and maintaining a robust protective barrier against harmful agents in the intestinal environment (1). The intestinal epithelium maintains cellular connections through tight junctions, which serve as a barrier to inflammation and infection-related cavity inflammation molecules (2). The tight junction barrier in the intestinal mucosa is regulated in response to the triggers of physiology and immunity, playing a vital role in maintaining immune balance (3). Pro-inflammatory cytokines, lipopolysaccharides, or abnormal conditions can cause the disruption of the tight junction barrier, triggering robust immune system stimulation and resulting in chronic inflammation in tissues (4). Moreover, these disruptions are increasingly recognized as key factors in disease development.

Mitophagy is a regulatory mechanism for the maintenance of a healthy population of mitochondria (5). Mitochondria are selectively sequestered into isolation membranes to form autophagosomes, which then fuse with lysosomes and eventually are removed from the cells, a process known as mitophagy (6). Recent studies have indicated the crucial contribution of mitophagy in preserving the structural integrity of the gut epithelial barrier. Zhao, H. et al. revealed that mitophagy is involved in maintaining both homeostasis and intestine repair, supporting the function of the intestinal barrier by regulating tight junctions in response to cellular stress (7). The dysregulation of mitophagy leads to an aberrant buildup of dysfunctional mitochondria and the generation of mitochondrial reactive oxygen species (ROS), which in turn triggers exaggerated inflammation, inflicting tissue injury, and ultimately escalating mortality rates (8). These detrimental effects are considered danger signals within the body. The amp-activated protein kinase (AMPK) is not only a critical energy sensor, but also a trigger of mitophagy (9). A recently published study revealed that andrographolide treatment induced parkin-dependent mitophagy in microglia, resulting in the removal of dysfunctional mitochondria and the inhibition of NLRP3 inflammasome activation (10). Besides, curcumin has been shown to protect intestinal barrier function and stimulate mitophagy through the AMPK-TFEB signal pathway (11). Therefore, the activated AMPK signaling pathway to enhance the mitophagy level holds promise as a promising approach for addressing intestinal barrier dysfunction.

Luteolin (LUT), a flavonoid compound widely present in natural plants, has been reported to exhibit a diverse array of biological properties, including anti-inflammatory, antioxidant, anti-apoptotic and various other beneficial effects (12). Numerous studies have demonstrated that LUT has therapeutic effects on various diseases through different mechanisms. Thaise Boeing et al. revealed that LUT alleviates irinotecan-induced oxidative stress by decreasing the levels of ROS, Lipid hydroperoxide (LOOH) and increasing endogenous antioxidants (13). Additionally, Robert Domitrović has shown that LUT inhibited inflammation by reducing the expression of nuclear factor NF-κB, Tumor Necrosis Factor-alpha (TNF-*α*) and Cyclooxygenase-2(COX-2) and apoptosis by reducing the expression of Caspase3 and Protein 53(p53), thereby alleviating cisplatin-induced nephrotoxicity (14). However, whether LUT can prevent LPS-induced intestinal damage remains unknown. Moreover, further studies are needed to explore the underlying mechanisms by which LUT regulates intestinal homeostasis. In the current investigation, a cellular model of lipopolysaccharide (LPS)-induced intestinal dysfunction was established with porcine jejunal epithelial cells (IPEC-J2), and an AMPK siRNA experiment was conducted to explore whether the shAMPK pathway is involved in the protective effects of LUT against LPS-induced intestinal dysfunction.

## 2 Materials and methods

### 2.1 Reagents

We purchased phosphate-buffered saline (PBS), fetal bovine serum (FBS), Dulbecco’s Modified Eagle’s Medium (DMEM) and the necessary antibiotics for cell culture from Gibco (Thermo Fisher Scientific, USA). LPS was sourced from Sigma-Aldrich (St Louis, United States) and LUT was purchased from Selleckchem (Houston, United States). The AMPK, p-AMPK, Parkin, Pink1, Claudin-1, Occludin, ZO-1 and GAPDH were provided from Proteintech (Wuhan, China).

### 2.2 Cell culture

The porcine intestinal epithelial cells (IPEC-J2) were generously supplied from the Laboratory of Hunan Agricultural University. A medium called high glucose DMEM, supplemented with 10% FBS, was utilized to culture the cells. These cells were grown in an environment with a constant temperature of 37°C, controlled humidity, and 5% CO_2_.

### 2.3 Establishment of AMPK-knockdown IPEC-J2 cell line

Stable knockdown of AMPK in the IPEC–J2 cell line was established based on our previous studies. The 293T cell line was used as the transfection tool cell. After resuscitating and culturing 293 T cells, a total of 3 μg of DNA plasmid was prepared. After mixing, the mixture was incubated statically on ice for 20 min, and then 200 μL of DMEM, 3 μL of Lip2000, and 1 μL of green fluorescent protein PCMV–EGFP were added drop–by–drop to the cells. IPEC–J2 cells were treated with shAMPK for 10 h, and then the medium was replaced with the cellular nutrient solution. After 24 h, the transfection efficiency was observed by fluorescence microscopy. Cell supernatants (lentivirus) were collected at 48 h and 72 h, respectively. The transfection process of the enveloped cell lentivirus was carried out in the same way to construct a shAMPK cell line and a control cell line (SC). We used Primer Blast to design short hairpin RNAs (shRNAs) specifically targeting AMPK, which were subsequently inserted into the lentiviral vectors. IPEC - J2 cells were then infected with these modified lentiviruses, and AMPK expression was then detected by fluorescence quantitative PCR. The sequences of the shRNAs used are shown in Table 1.

**Table 1:** Sequence of short-hairpin RNA.

Gene name	Primer sequences
shAMPKF	CCGGTTTCAGGCATCCTCATATAATCTCGAGATTATATGAGGATGCCTGAAATTTTTG
shAMPKR	AATTCAAAAATTTCAGGCATCCTCATATAATCTCGAGATTATATGAGGATGCCTGAAA

### 2.4 Cell viability assay

The CCK-8 assay kit (Bimake, USA) was used to assess cell viability, following the specifications from the manufacturer. A seeding concentration of 6 × 10^4^ cells per well was used for the cells in a 96-well plate. Subsequently, the cells were exposed to varying concentrations of LPS or LUT for a duration of 24 h. In the co-culture experiment, LUT was administered 6 h prior to the addition of LPS. After the specified incubation period, the assessment of cell viability was carried out using the CCK-8 assay.

### 2.5 Determination of contents of MDA, and activities of CAT and SOD

After the cells were treated, malondialdehyde (MDA) content, along with the enzymatic levels of catalase (CAT) and superoxide dismutase (SOD) was determined in the lysed cells of each group using assay kits obtained from the respective manufacturers (Jiancheng, Nanjing, China) and following the provided instructions.

### 2.6 Assessment of cellular ROS levels

The cellular reactive oxygen species (ROS) levels were determined by using a ROS-sensitive fluorescence indicator (Beyotime, China). In brief, the cells were treated with PBS before being incubated with a ROS-sensitive fluorescence indicator. Subsequently, the cells were then analyzed using a flow cytometer to measure the fluorescence signal emitted by the ROS indicator. The intensity of the fluorescence signal was used to determine the cellular ROS levels.

### 2.7 Immunofluorescence observation

The IPEC–J2 cells were exposed to paraformaldehyde to preserve their structure. Then, they were washed with PBS and blocked with BSA. Next, the cells were exposed with primary antibodies targeting Occludin. Afterwards, remove the cells were subjected to incubation with a secondary antibody that was labeled with Alexa-Fluor-555. Lastly, the cells were stained with DAPI, and the staining was observed using immunofluorescence microscopy.

### 2.8 Cell apoptosis assay

Cell apoptosis was assessed using the Annexin V-FITC/PI kit (Beyotime Biotechnology, China) and detected by flow cytometry according the manufacture’s instruction.

### 2.9 Observation of mitochondrial ultrastructure

The IPEC-J2 cells underwent a series of steps including fixation, dehydration, embedding in epoxy resin, slicing into thin sections, staining, and subsequent observation of their ultrastructure under a transmission electron microscope.

### 2.10 Measurement of mitochondrial membrane potential (MMP)

The IPEC-J2 cells were incubated and then stained with a fluorescent dye called JC-1 (Beyotime Biotechnology, China). The fluorescence emitted by the dye was then measured using the FITC and PE channels of a BD Accuri C6 flow cytometer to determine the change in MMP.

### 2.11 Quantitative real-time PCR (qPCR) analysis

The TransZol-Up reagent was utilized to obtain total RNA from IPEC-J2 cells. Subsequently, the RNAs was reverse transcribed into cDNA for qPCR. Gene-specific primers were designed using Primer Premier software, and their sequences are presented in Table 2. To evaluate the relative expression levels, the 2^−ΔΔCT^ method was used, with *β*-actin as the reference gene. Our analysis provided insights into the fold changes of mRNA expression for each gene of interest.

**Table 2:** Primer sequence.

Gene name	Primer sequences (5′ to 3′)
β-actin	Forward: CTGACCCTGAAGTACCCCAT
	Reverse: TGTCATCTTCTCTCTGTTGGCTT
IL-6	Forward: TGAGGCAAAGGGAAAGAATCC
	Reverse: CAGGTGCCCCAGCTACATTAT
IL-1β	Forward: CCTGGACCTTGGTTCTCT
	Reverse: GGATTCTTCATCGGCTTCT
NF-κB	Forward: GGGGACTACGACCTGAATGC
	Reverse: CTCCCCGAGTTCCGATTCAC
Bcl-2	Forward: GGATAACGGAGGCTGGGATG
	Reverse: TTATGGCCCAGATAGGCACC
Bax	Forward: GCCCTTTTGCTTCAGGGTTTC
	Reverse: CAATGCGCTTGAGACACTCG
Caspase-3	Forward: TGTGGGATTGAGACGGACAG
	Reverse: TCCGTCCTTTGAATTTCGCC
Caspase-9	Forward: GGCCAGTGGACATTGGTTCT
	Reverse: GGCCTTGGCAGTCAGGTT
AMPK	Forward: AAATCGGCCACTACATCCTGG
	Reverse: TCATGTTTGCCAACCTTCACT
ULK1	Forward: CCTGTGACACCGACGACTTT
	Reverse: CAGTGAGCTCCCGCTGC
Pink	Forward: CAGGGCGGTGATTGACTACA
	Reverse: GGTTGGAGAGCCCGAAGATT
Parkin	Forward: CCAAACCGGATGAGTGGTGA
	Reverse: GGTCATTGAGAATCGTCACA

### 2.12 Western blot analyses

The cells were lysed using a protein extraction-optimized buffer, and the concentration of the resulting proteins was determined using the BCA protein assay kit. The total protein extract was then separated based on their molecular weight using SDS-PAGE. Subsequently, the separated proteins were transferred onto a PVDF membrane. The membrane was successively exposed to the primary antibody, diluted according to recommendations, and then to the appropriate secondary antibody. To visualize the presence of specific proteins, an enhanced chemiluminescence system was employed. To visualize the presence of specific proteins, an enhanced chemiluminescence system was employed. The protein levels of GAPDH, AMPK, P-AMPK, Caspase3, Cleaved-Caspase3, Bax, Bcl-2, Parkin, Pink1, ULK1, ZO-1, Claudin1, and Occludin were determined by Western blot analysis. The primary antibodies used in this study were AMPK (1:2000, Proteintech, China), P-AMPK (1:2000, Proteintech, China), Caspase3 (1:1000, Proteintech, China), Cleaved-Caspase3 (1:1000, Proteintech, China), Bax (1:500, Wanleibio, China), Bcl-2 (1:500, Wanleibio, China), Parkin (1:1000, Wanleibio, China), Pink1 (1:1000, Wanleibio, China), ZO-1 (1:1000, Bimake, American), Claudin1 (1:1000, Bimake, American)and Occludin (1:1000, Bimake, American). The anti-rabbit and anti-mouse secondary antibodies were purchased from Proteintech, China. Protein bands were imaged by Image Lab Software (Bio-Rad, USA) and analyzed by ImageJ software. The GAPDH (1:5000, Proteintech, China) band was used as a control to perform standardized quantitative analysis on Western blots.

### 2.13 Statistical analysis

The data are presented as the mean ± SD. One–way analysis of variance (ANOVA) was used to evaluate group differences, and then Duncan multiple tests were conducted to determine the *p* values. Statistical significance was defined as a *p*-value of less than 0.05. “*” denotes a significant difference compared with the control group (“*” *p* < 0.05; “**” *p* < 0.01), and “#” denotes a significant difference compared with the LPS group (“#” p < 0.05; “##” p < 0.01).

## 3 Results

### 3.1 Concentration-dependent effects of LPS and LUT on cell viability

**Figure 1:**
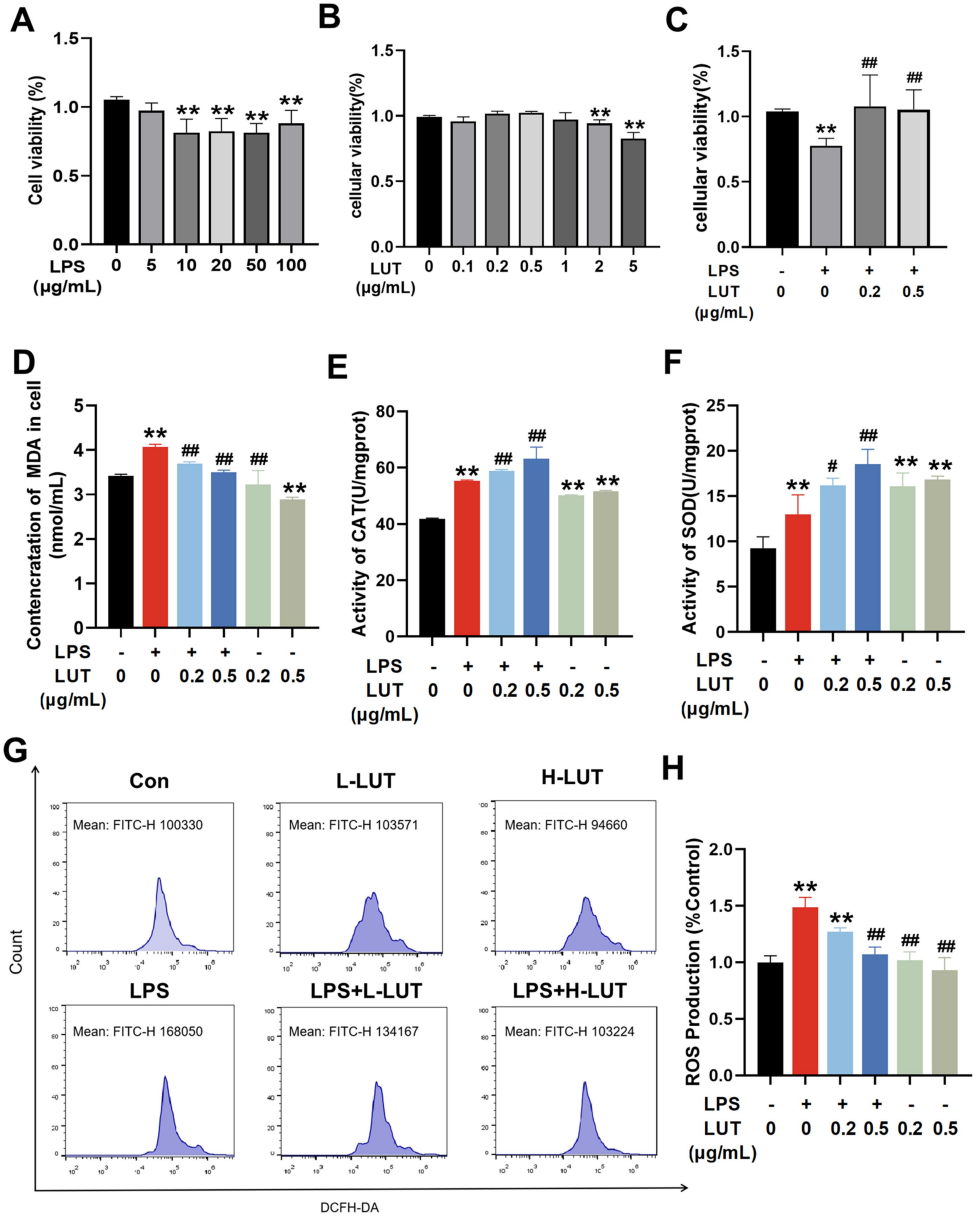
Effects of Luteolin (LUT) on cell viability and antioxidant capacity in LPS-induced IPEC-J2 cells. **(A)** Cells were exposed to various concentrations of LPS (0–100 μg/mL) for 12 h. **(B)** Cells were treated with various concentrations of LUT (0–0.5 μg/mL) for 12 h. **(C)** Cells were exposed to different concentrations of LUT for a duration of 8 h, and subsequently treated with 10 μg/mL LPS for an additional 12 h. **(D)** MDA concentration. **(E)** CAT activity. **(F)** SOD activity. **(G,H)** ROS production. Data are presented as the means ± SD. “*” Indicates significant difference compared with control group (“*” *p* < 0.05; “**” *p* < 0.01), “#” indicates significant difference compared with LPS group (“#” *p* < 0.05; “##” *p* < 0.01). “L-LUT” indicates concentrations of 0.2 μ g/mL for LUT, “H-LUT” indicates concentrations of 0.5 μg/mL for LUT. Below is the same.

To select optimal concentrations for further research, we initially assessed the viability of IPEC - J2 cells through treatment with different concentrations of LPS or LUT. In Figure 1, we identified that LPS treatment at concentrations exceeding 10 μg/mL led to a marked reduction in cell viability after 12 h compared with the control group (*p* < 0.01). In the 5 μg/mL LPS–treated group, the cell viability of IPEC–J2 was decreased compared with the control group, and the difference was not significant (*p* > 0.05) (Figure 1A). LUT concentrations of 0.2 μg/mL and 0.5 μg/mL showed an increasing trend in cell viability compared with the control (0 μg/mL LUT), but the increase was not significant (*p* > 0.05). However, when the dose of LUT exceeded 1 μg/mL, the viability of IPEC–J2 cells declined compared with the control (0 μg/mL LUT). Significant differences were observed at doses of 2 μg/mL and 5 μg/mL (*p* < 0.01) (Figure 1B). Notably, pretreatment with LUT at concentrations of 0.2 μg/mL or 0.5 μg/mL effectively mitigated the decrease in cell viability induced by 10 μg/mL of LPS (*p* < 0.01) (Figure 1C). Consequently, for subsequent experiments investigating the beneficial effects of LUT against LPS-induced oxidative stress in IPEC-J2 cells, we selected concentrations of 0.2 μg/mL and 0.5 μg/mL for LUT, in combination with 10 μg/mL for LPS.

### 3.2 LUT elevates antioxidant ability and reduction ROS generated in in LPS treated IPEC-J2 cells

To explore the impact of LUT on LPS-induced oxidative stress damage in IPEC-J2 cells, the oxidative stress was conducted to assess the levels of MDA, activation of antioxidant enzymes (SOD and CAT), and intracellular ROS. The results showed that the contents of intracellular antioxidant enzymes (SOD, CAT) and MDA in IPEC-J2 cells in the LPS (10 μg/mL) group were significantly increased compared with those in the Con group (*p* < 0.01). Treatment with LUT led to a significant reduction in MDA content in cells exposed to LPS, as well as in cells without LPS treatment (*p <* 0.01) (Figure 1D). Moreover, LUT treatment notably increased the activities of SOD and CAT (*p <* 0.05) (Figures 1E,F). The ROS levels were substantially elevated in cells treated with LPS compared to the control group. However, pretreatment with LUT dose-dependently decreased the ROS levels in comparison to the LPS treatment (*p <* 0.05) (Figures 1G,H). These results suggest that LUT administration exhibits antioxidant properties by reducing MDA content, enhancing antioxidant enzyme activation, and attenuating the generation of ROS in LPS-treated IPEC-J2 cells.

### 3.3 LUT attenuates the LPS-induced tight junction injury in IPEC-J2 cells, decreases pro-inflammatory cytokine expression in LPS-stimulated IPEC-J2 cells

**Figure 2:**
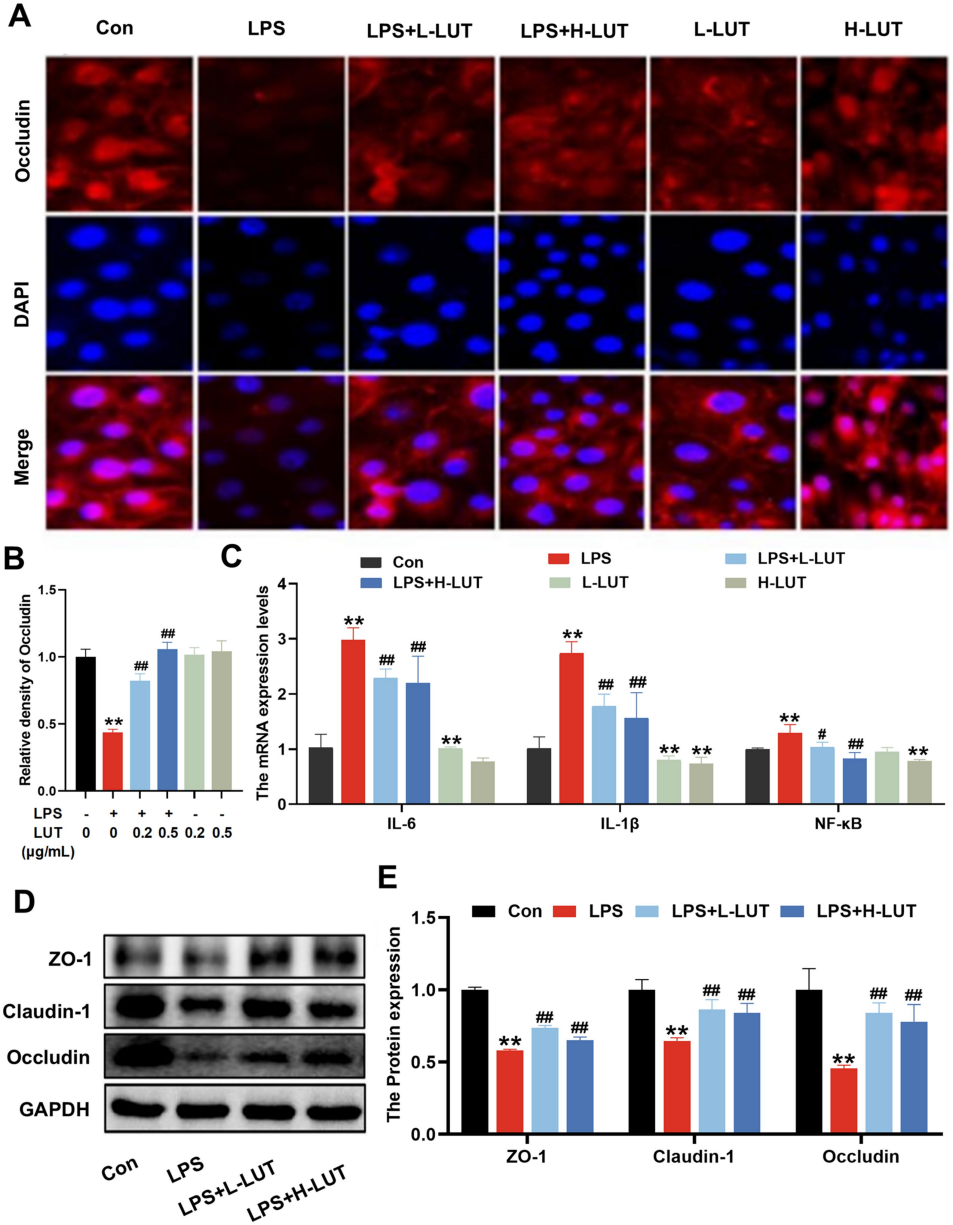
Effects of LUT on tight junction proteins expression and proinflammatory cytokine expression in LPS-induced IPEC-J2 cells. **(A,B)** The Fluoresce expression of Occludin. **(C)** The mRNA expression levels of IL-6, IL-1*β* and NF-κB. **(D)** Western blot analysis of ZO-1, Claudin-1 and Occludin. **(E)** Quantitation of protein bands of ZO-1, Claudin-1 and Occludin. “*” indicates significant difference compared with control group (“*” *P* <0.05; “**” *P* <0.01), “#” indicates significant difference compared with LPS group (“#” *P* <0.05; “##” *P* <0.01).

As shown in Figure 2, the intensity of red fluorescence on the membrane surface of IPEC–J2 cells in the LPS group decreased significantly compared with that in the Con group (*p* < 0.01). Compared with the LPS group, cells co - treated with Lut (0.2 μg/mL and 0.5 μg/mL) and LPS showed a significant increase in surface red - fluorescence intensity (*p* < 0.01). The fluorescence intensity on the surface of IPEC–J2 cell membranes was stronger at a LUT concentration of 0.5 μg/mL. In addition, the intensity of IPEC–J2 cell membrane surface fluorescence in the LUT (0.2 μg/mL and 0.5 μg/mL) treatment groups showed a linear distribution of red fluorescence on the surface of the cell membranes when compared with that in the Con group (*p* > 0.05) (Figures 2A,B). To investigate the impact of LUT on the LPS–induced inflammatory response in IPEC–J2 cells, we assessed the expression of pro–inflammatory cytokines. RT–qPCR was used to measure the gene expression levels of pro–inflammatory cytokines. As shown in Figure 2C, LPS treatment resulted in a notable up - regulation of the relative mRNA expression levels of IL - 6, IL-1β, and NF-κB. However, Co-treatment with LUT (0.2 μg/mL and 0.5 μg/mL) effectively reversed the mRNA expression levels of IL-6, IL-1β and NF-κB (*p* < 0.01), and the effect of 0.5 μg/mL LUT was more significant than that of 0.2 μg/mL LUT. Treatment with LPS significantly reduced the protein expression levels of tight–junction proteins ZO-1, Claudin-1, and Occludin in IPEC - J2 cells compared with the control group. However, pretreatment with LUT mitigated the reduction of ZO-1, Claudin-1, and Occludin protein expression. In the L-LUT treatment group, the rise was even more significant (Figures 2D,E).

### 3.4 LUT attenuates the LPS-induced IPEC-J2 cells apoptosis by inhibiting mitochondrial mediated apoptotic pathway

**Figure 3:**
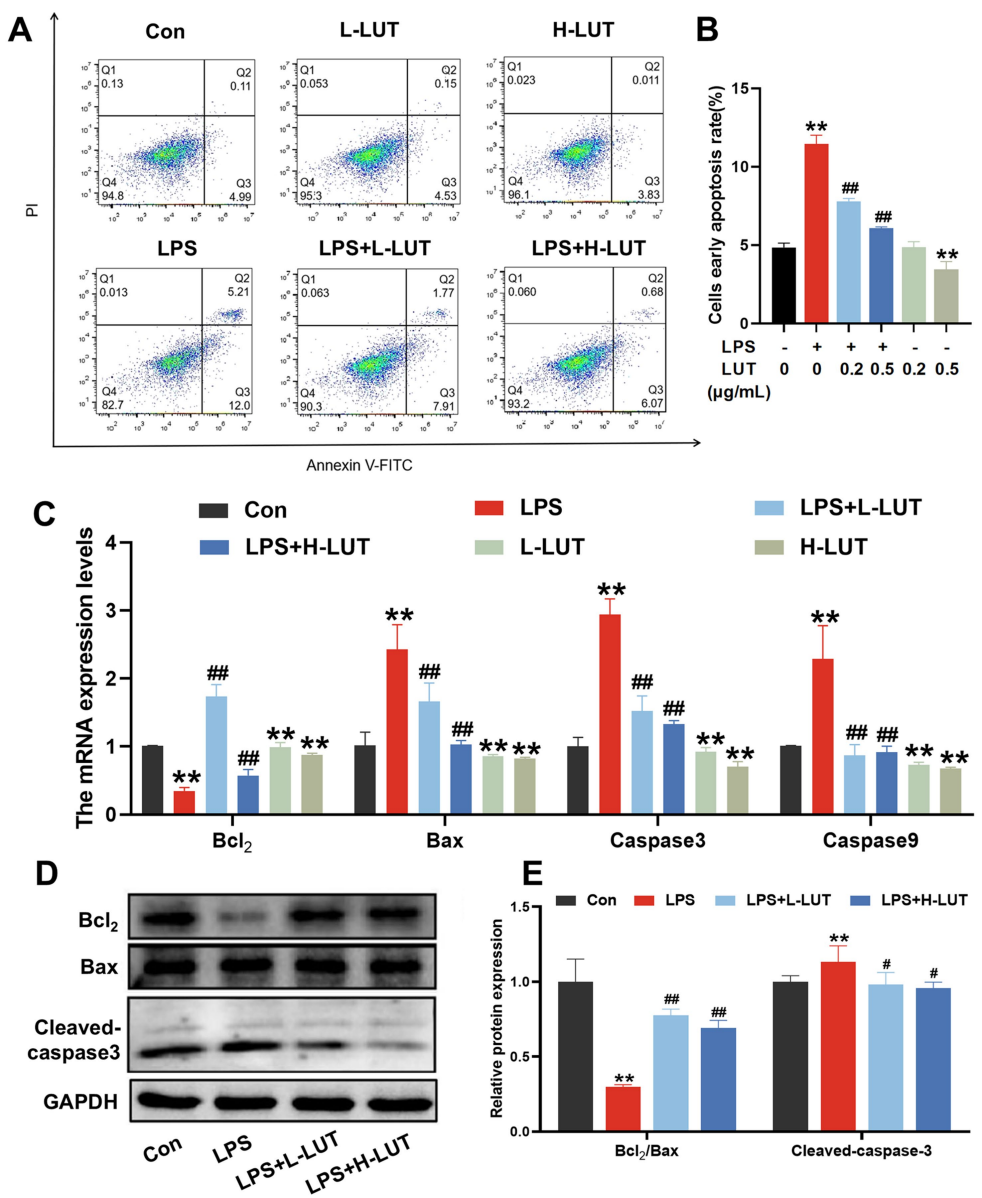
Effects of LUT on apoptosis in LPS-induced IPEC-J2 cells. **(A,B)** Apoptosis cells rate. **(C)** The mRNA expression levels of Bcl-2, Bax, Caspase-3 and Caspase-9. **(D)** Western blot analysis of Bcl-2, Bax and Cleaved-caspase3. **(E)** Quantitation of protein bands of Bcl-2/Bax and Cleaved-caspase3. “*” indicates significant difference compared with control group (“*” *P* <0.05; “**” *P* <0.01), “#” indicates significant difference compared with LPS group (“#” *P* <0.05; “##” *P* <0.01).

Apoptotic cells were analyzed by Annexin V-FITC/PI kit and flow cytometry to visualize and quantify. As shown in Figures 3A,B, in comparison to the control group, LPS treatment significantly increased the IPEC-J2 cell early apoptosis rate, whereas LUT pretreatment inhibited the increase of cell early apoptosis rate (*p* < 0.01). Additionally, high concentration of LUT treatment also inhibited the IPEC-J2 early apoptosis rate (*p* < 0.01). Moreover, LPS treatment effectively decreased the expression of Bcl-2 mRNA and increased the expression of Bax, Caspase-3, and Caspase-9 mRNA, whereas LUT pretreatment dramatically reversed the change of the above-mentioned indicators (*p* < 0.01) (Figure 3C). Furthermore, LPS treatment effectively decreased the ratio of Bcl-2/Bax mRNA and increased the expression of Caspase-3 and Caspase-9 mRNA, whereas LUT pretreatment dramatically reversed these changes (p < 0.01). The effect was more pronounced in the H-LUT treatment group (Figure 3C). Furthermore, LPS treatment significantly decreased the ratio of Bcl-2/Bax and increased the activation of caspase-3 compared with the control group, as determined by Western blot assay. Similarly, LUT pretreatment reversed these changes (*p* < 0.05). Among them, the L-LUT-treated group showed a higher rise in the ratio of Bcl2/Bax protein expression in cells compared to the LPS group (*p* < 0.01) (Figures 3D,E).

### 3.5 LUT mitigates LPS-induced IPEC-J2 cell barrier dysfunction by enhancing mitophagy and activating AMPK

**Figure 4:**
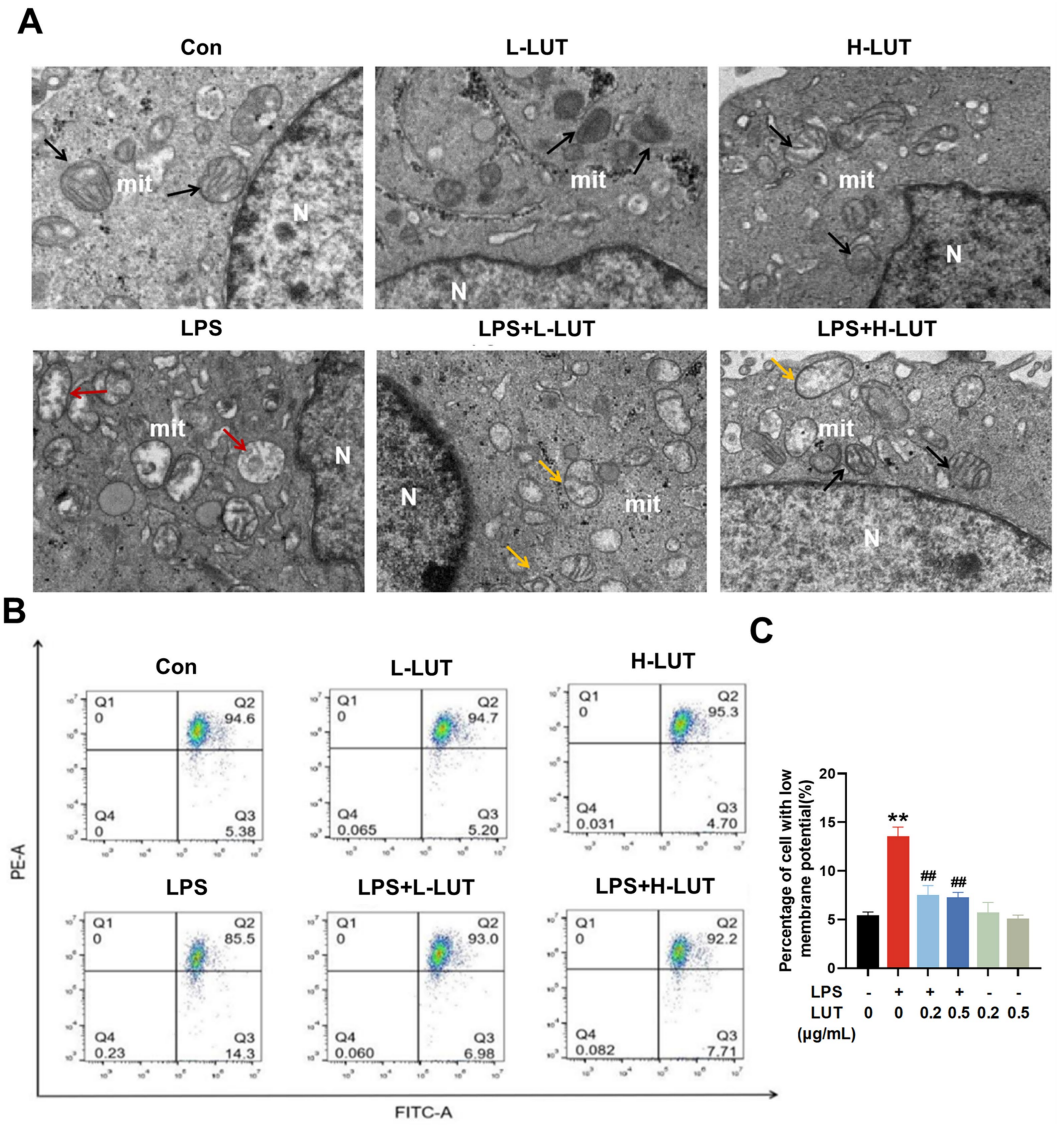
Effects of LUT on mitochondrial damage in LPS-induced IPEC-J2 cells. **(A)** Ultrastructure of mitochondria (N: Nucleus; mit: mitochondria; Black arrows represent the normal mitochondria; Red arrows represent the damaged mitochondria; Yellow arrows represent mitochondria engulfed by autophagosomes); **(B,C)** Mitochondrial membrane potential (MMP). “*” indicates significant difference compared with control group (“*” *P* <0.05; “**” *P* <0.01), “#” indicates significant difference compared with LPS group (“#” *P* <0.05; “##” *P* <0.01).

**Figure 5:**
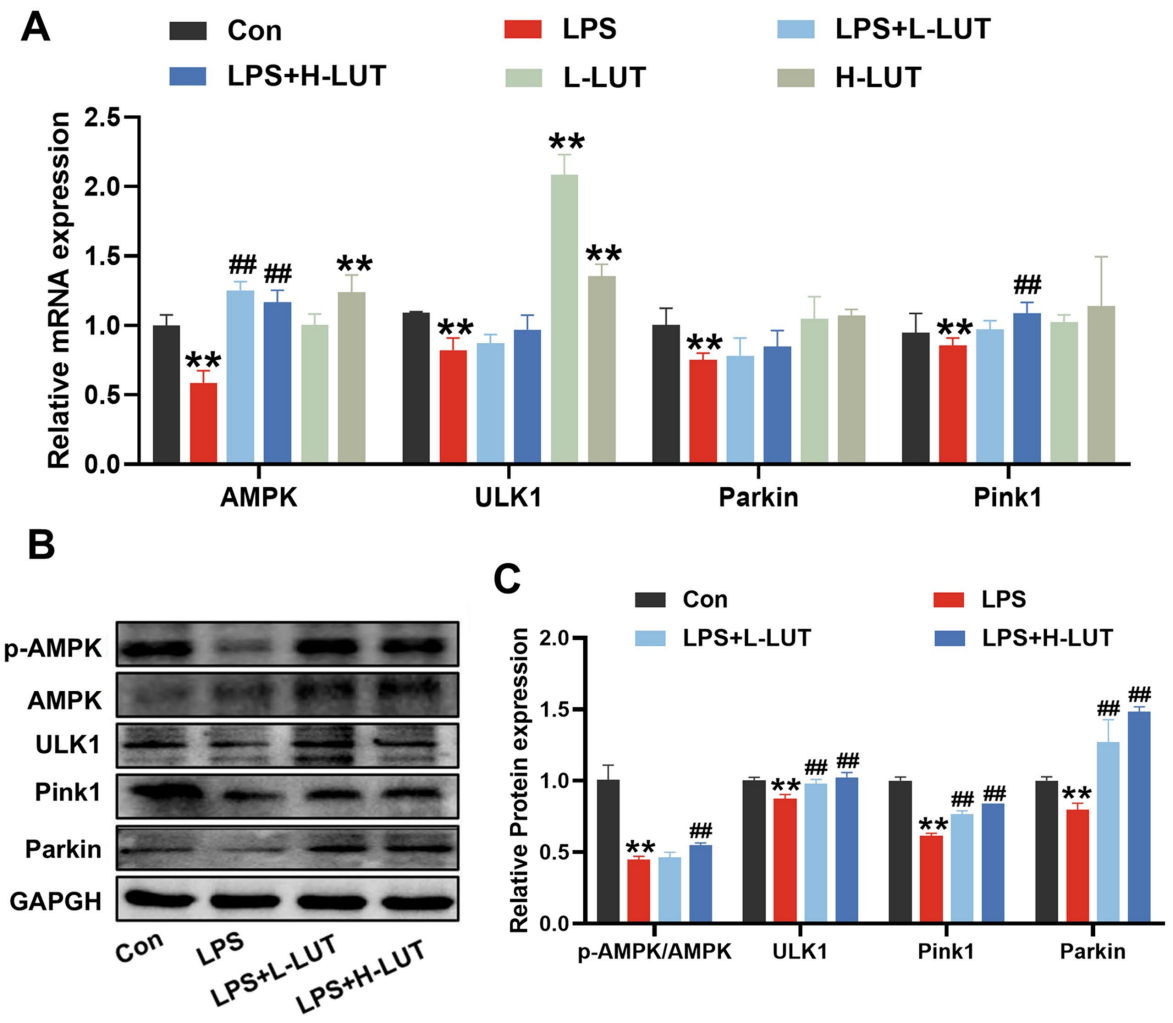
Effects of LUT on the expression levels of AMPK and mitophagy-related genes in LPS-induced IPEC-J2 cells. **(A)** The mRNA expression levels of AMPK, ULK1, Parkin and Pink1. **(B)** Western blot analysis of p-AMPK, AMPK, ULK1, Parkin and Pink1. **(C)** Quantitation of protein bands of p-AMPK, ULK1, Parkin and Pink1. “*” indicates significant difference compared with control group (“*” *P* <0.05; “**” *P* <0.01), “#” indicates significant difference compared with LPS group (“#” *P* <0.05; “##” *P* <0.01).

To explore the potential mechanism of LUT on LPS-induced IPEC-J2 cells, mitochondrial function and mitophagy were evaluated by analyzing mitochondrial ultrastructure, MMP, as well as the expression of mitophagy-related mRNAs and proteins. TEM observation showed that mitochondria showed swelling, membrane damage, and a limited presence of autophagosomes in LPS treated with IPEC-J2 cells. In contrast, normal mitochondrial morphology and a higher abundance of autophagosomes were display in the IPEC-J2 cells pretreated with LUT (Figure 4A). Additionally, The MMP in IPEC-J2 cells pretreatment of LUT was significantly increased compared with IPEC-J2 Cells treat with LPS (Figures 4B,C). Meanwhile, LPS treatment significantly down-regulated the mRNA expression of AMPK, ULK1, Parkin, and Pink1 compared with the control group (*p* < 0.01). Compared with the LPS-treated group, LUT pretreatment up-regulated the transcript levels of AMPK, with a significant difference (p < 0.01). Although LUT pretreatment also up-regulated the transcript levels of ULK1 and Parkin, the differences were not significant (*p* > 0.05). In contrast, H-LUT significantly up-regulated the transcript levels of Pink1 (*p* < 0.01) (Figure 5A). Furthermore, LPS treatment decreased the protein expression of p-AMPK/AMPK and autophagy-related proteins (ULK1, Parkin, Pink1). LUT pretreatment significantly increased all of the above metrics (*p* < 0.05), with a more pronounced increase observed in the H-LUT group than in the L-LUT group (Figures 5B,C).

### 3.6 AMPK silence inhibited the protective effects of LUT against LPS-induced tight junction damage and oxidative stress in IPEC-J2 cells

**Figure 6:**
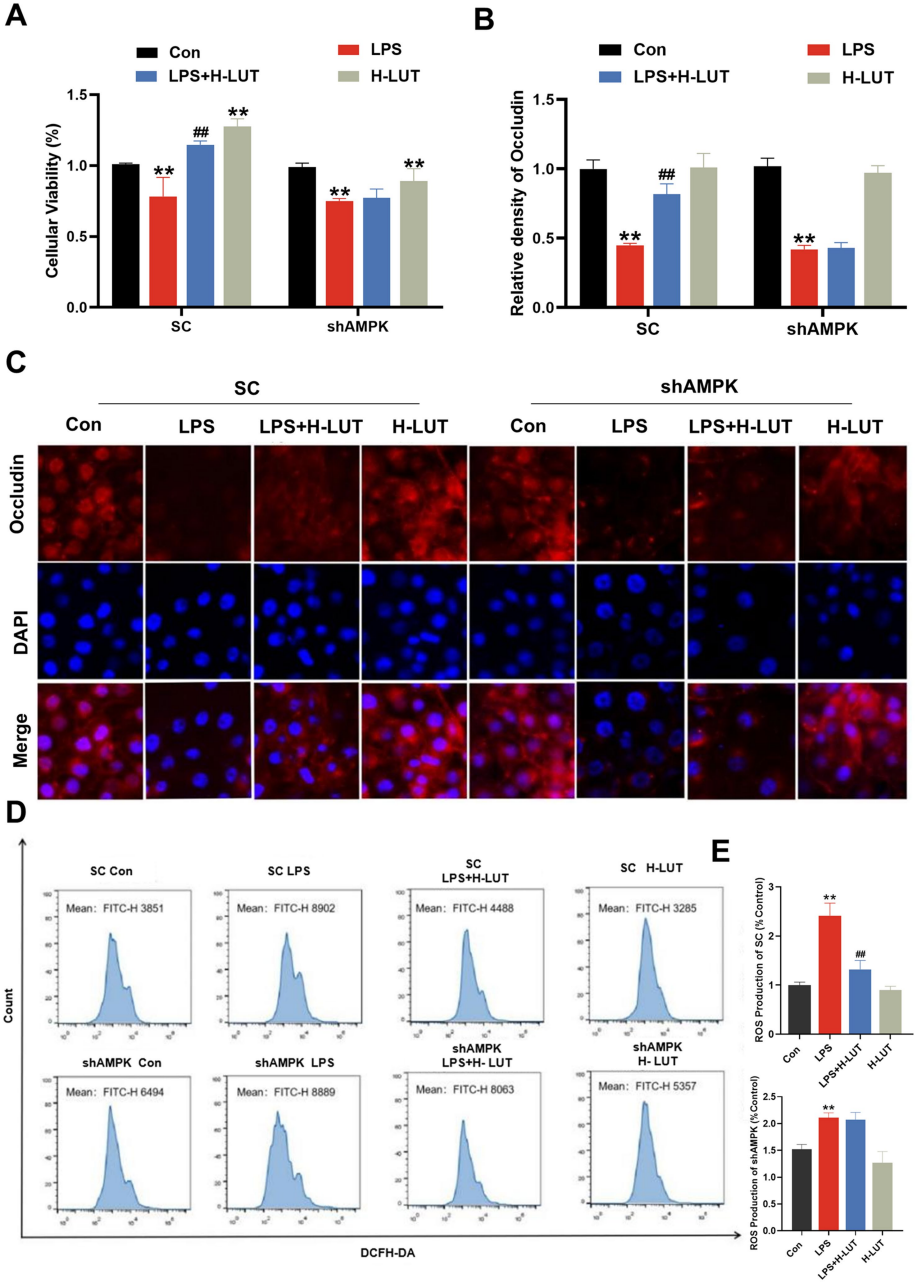
Effects of LUT on cell viability, tight junction protein expression and ROS gather in LPS-induced IPEC-J2 cells after AMPK knockdown. **(A)** Cell viability. **(B,C)** Occludin protein expression levels. **(D,E)** ROS production. “*” indicates significant difference compared with control group (“*” *P* <0.05; “**” *P* <0.01), “#” indicates significant difference compared with LPS group (“#” *P* <0.05; “##” *P* <0.01).

To investigate the role of AMPK in the cytoprotective effects of LUT against LPS–induced intestinal damage, small interfering RNA (siRNA) silencing of the shAMPK genes was undertaken. Then, IPEC–J2 cells transfected with control short hairpin RNA (SC) or shRNA against AMPK (shAMPK) were treated with LUT or LPS. The cellular viability and Occludin protein expression levels in the SC cell line exhibited concordance with the findings reported in prior studies. In the shAMPK cell line, the intensity of cell membrane red fluorescence was significantly reduced in the LPS–treated group compared with Con (*p* < 0.01), however, the fluorescence intensity of Occludin protein expression on the membrane surface was still lower in the group co-treated with LUT (0.5 μg/mL) and LPS compared with the LPS (10 μg/mL) group (*p* > 0.05). The cell viability and Occludin protein expression levels in cells treated with a combination of LUT and LPS demonstrated similarity to those in cells treated with LPS alone (Figures 6A–C). Additionally, Flow cytometry results showed that the ROS levels in the SC cell line were consistent with the previously reported results. However, in the shAMPK cell line, the ROS levels of LPS treatment were increased dramatically compared with control group, and the ROS levels of LUT pretreatment were not changed compared with LPS treatment (Figures 6D,E).

### 3.7 AMPK silencing blocked the enhancement of mitophagy by LUT in LPS-treated IPEC-J2 cells

**Figure 7:**
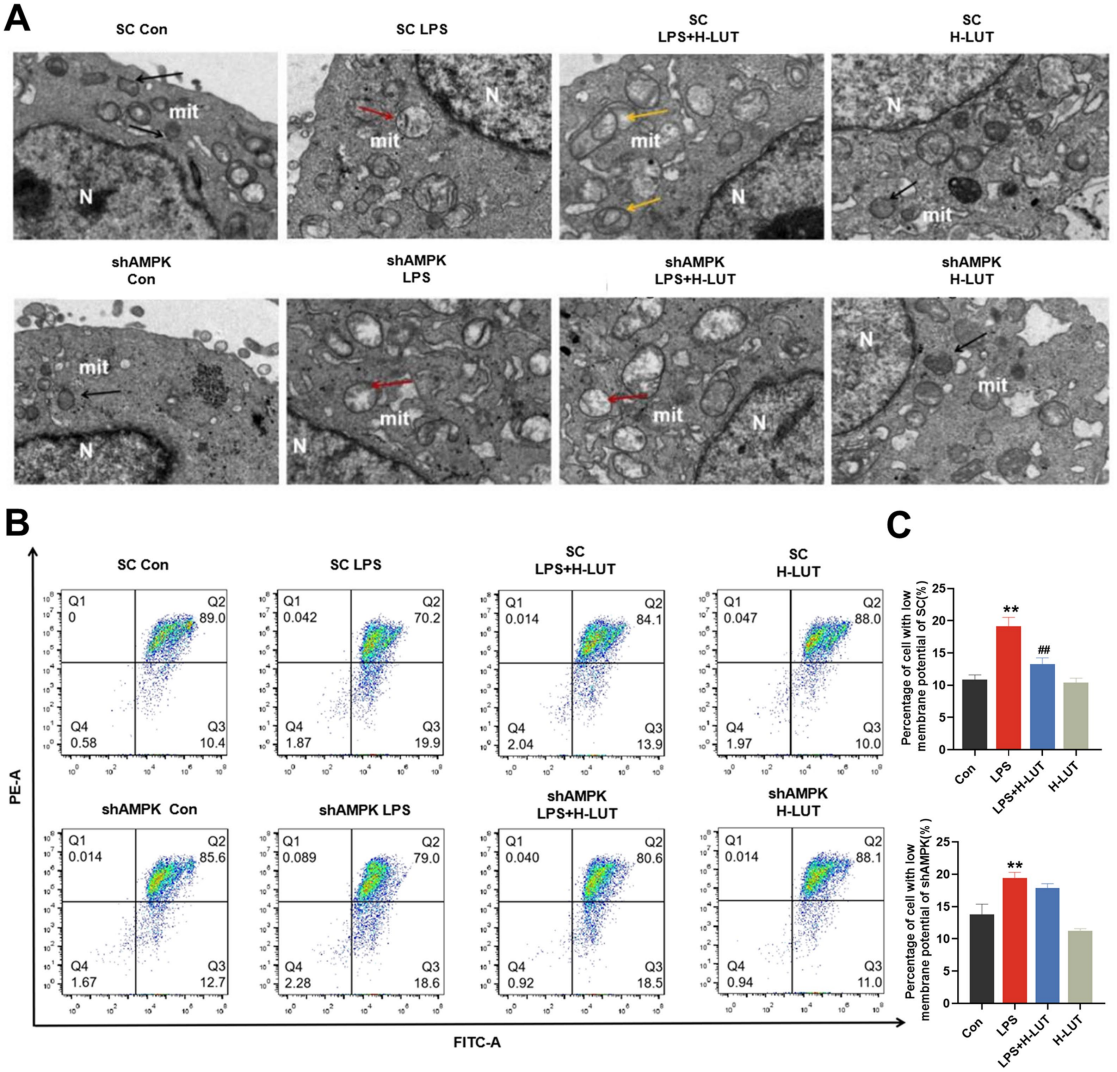
Effects of LUT on mitochondrial damage in LPS-induced IPEC-J2 cells after AMPK knockdown. **(A)** Ultrastructure of mitochondria. **(B,C)** MMP. “*” indicates significant difference compared with control group (“*” *P* <0.05; “**” *P* <0.01), “#” indicates significant difference compared with LPS group (“#” *P* <0.05; “##” *P* <0.01).

Transmission Electron Microscope (TEM) observed that morphology and structure of the mitochondria in the SC cell lines were consistent with the previously reported results. In the shAMPK cell line, the mitochondria were obvious damage (mitochondria with swelling, broken cristae and vacuolation), whereas only few autophagosomes were noticed in IPEC-J2 cells treated with LPS, and mitochondrial morphological structure of LUT pretreatment was similar with LPS treatment (Figure 7A). After AMPK gene silencing, the mitochondrial membrane potential of IPEC-J2 cells in the LPS (10 μg/mL) group was significantly reduced compared with that of the Con group; however, there was a tendency for the mitochondrial membrane potential of IPEC-J2 cells in the LUT (0.5 μg/mL) and LPS cotreated group to be elevated compared with that of the LPS (10 μg/mL) group, but it was not significant (*p* > 0.05). The measurement results of mitochondrial membrane potential also indicated that LUT did not exhibit a protective effect against the decline in mitochondrial membrane potential induced by LPS in shAMPK cell lines (Figures 7B,C).

## 4 Discussion

Luteolin, a naturally occurring flavonoid found in various plants, has potential applications in the development of functional foods due to its beneficial properties. Extensive research has demonstrated its diverse biological activities, including neuroprotective, cardioprotective, and anti-diabetic effects (15). Therefore, through establishing an infection model by inducing LPS-mediated damage in IPEC-J2 cells and conducting AMPK siRNA experiments, our findings revealed that LUT effectively attenuated porcine intestinal tight junction damage induced by LPS through enhancing mitophagy via shAMPK signaling pathway activation, highlighting its therapeutic potential for mitigating intestinal damage.

The intestinal epithelial barrier represents the primary defense mechanism against a diverse array of pathogens and is integral in preserving intestinal mucosal homeostasis (16). Crucial to the maintenance of the intestinal epithelial barrier are tight junctions, intricate multi-protein complexes composed of various proteins including ZO-1, Claudin-1, and Occludin (17). Numerous investigations have demonstrated that LPS can induce damage to tight junctions in the intestine, thereby augmenting epithelial permeability (18). Our previous studies have shed light on the pivotal role played by LUT in safeguarding intestinal health. Notable effects of LUT include the promotion of beneficial microorganisms and the inhibition of harmful microorganisms as well as the improvement of intestinal development and the enhancement of overall health in broiler chickens and weaned piglets (19, 20). We found that LPS treatment of IPEC - J2 cells significantly increased the expression levels of CAT and SOD, which possibly may be related to the self - protection mechanism of cells under stress. Furthermore, LUT has been observed to attenuate the inflammatory response provoked by intestinal stress induced by LPS and oxidative stress. In the present study, we confirmed that LUT could mitigate oxidative stress damage and alleviate inflammatory response in LPS-induced IPEC-J2 cells, thus improving the intestinal barrier function in IPEC-J2 cells. These findings were supported by a recent study proving that LUT could improve the intestinal mucosal barrier by enhancing intestine barrier function through its antioxidant and anti-inflammatory properties intestinal mucositis (21).

Recently studies have demonstrated that LPS-induced cellular oxidative stress is known to inflict significant damage to mitochondria and endoplasmic reticulum, consequently impeding proper functioning of mitophagy. Next, we investigated the potential protect mechanism of LUT against LPS-induced intestinal damage. Mitophagy serves as a pivotal mechanism for maintaining mitochondrial quality control by removing damaged mitochondria and mitigating reactive oxygen species (ROS) production (22). The intricate link between mitochondrial oxidative stress, ROS generation, and mitophagy is implicated in the pathogenesis of intestinal barrier dysfunction and other related pathological conditions (23). Emerging evidence suggests that mitophagy involving Parkin/Pink1 signaling pathway activation is tightly regulated by ROS (24). Xiao Li et al. revealed that excess ROS and mitochondrial fragmentation accompanied with the reduction of Pink1 and Parkin expression and the increase of apoptosis in the tubular cells of db/db mice (25). In this study, the occurrence of LPS - induced apoptosis in IPEC - J2 cell injury had a negative influence. LUT treatment in this study effectively inhibited the occurrence of LPS - induced apoptosis. The results of this study showed that compared with the control group, the LPS group significantly increased the transcription levels of caspase3, caspase9 and Bax genes and inhibited the expression level of Bcl2 gene; the protein results showed that the Bcl2/Bax ratio was significantly reduced. LUT treatment could effectively inhibit the occurrence of apoptosis. This suggests that LPS can induce IPEC - J2 cells to undergo programmed cell death and a series of other response mechanisms, resulting in cellular inflammatory damage, oxidative damage, and intestinal barrier damage. LUT can inhibit LPS - induced apoptosis in IPEC - J2 cells and thus play a protective role. In summary, LUT attenuates LPS - induced intestinal damage possibly through a combination of multiple mechanisms including anti-inflammatory, antioxidant, protecting intestinal barrier function and anti-apoptotic. In present study, we further observed that that LUT treatment effectively mitigated oxidative stress, restored mitophagy function, and improved autophagy. These findings contribute to the growing evidence supporting the involvement of LUT in LPS-induced intestinal injury through its interaction with the AMPK-mediated mitochondrial autophagy signaling pathway, particularly the Parkin/Pink1 pathway.

AMPK plays a crucial role in the regulation of mitochondrial homeostasis, including the process of mitophagy (26). AMPK interacted with substructure ULK1 was recently considered to be initiation of the post-transcription signals of mitophagy (27). PINK1 clusters on the outer membrane of dysfunctional mitochondria, where it induces and enhances the activity of Parkin’s E3 ubiquitin ligase (28). This activation subsequently triggers autophagy, or mitophagy. Recent research has shown that curcumin helps preserve intestinal barrier function and promotes mitophagy through the AMPK signal pathway (11). Recently studies reported that AMPK-PINK1/Parkin pathway-mediated mitophagy is essential for mitigating oxidative stress-induced the damage of intestinal epithelial barrier and the dysfunction of mitochondrial energy metabolism in IPEC-J2 cells (29). Accumulating evidences have linked impaired mitophagy to intestinal diseases, indicating the protective role of mitophagy (30). Huang Y et al. revealed that Suppression or disruption of cellular mitophagy induced by indoxyl sulfate has been linked to intestinal barrier impairment (31). Our findings reveal that LPS exert an inhibitory effect on the AMPK signaling pathway, resulting in impaired mitophagy autophagy, intestinal barrier disruption, and cellular apoptosis. Conversely, treatments with LUT could enhance mitophagy via AMPK/ULK pathway, as evidence by activated AMPK signals by the up-regulation of Parkin/Pink1. Similar results were reported in once recently by Yu et al., who found that quercetin, similar to LUT, belongs to the class of flavonoids and could ameliorates Ethanol-Induced Hepatic Mitochondrial Damage via the promotion of AMPK-mediated hepatic mitophagy (32). In the present study, we further knockdown of AMPK by shRNA, the protect effects of LUT against LPS-induced IPEC-J2 cells damage, as evidenced by the accumulation of excessive ROS and impaired mitophagy. Take together, these results indicated that LUT attenuates LPS-induced damage in IPEC-J2 cells by enhancing mitophagy via AMPK signaling pathway activation. These findings highlighting the crucial role of AMPK in mediating the therapeutic potential of LUT and regulating oxidative stress and cellular homeostasis.

## Data Availability

The raw data supporting the conclusions of this article will be made available by the authors, without undue reservation.
